# Supplementary material for: Perfect circular polarization of elastic waves in solid media
Source: Nat Commun. 2024 Feb 12;15:992. doi: 10.1038/s41467-024-45146-w (PMC10861468; doi:10.1038/s41467-024-45146-w)
Supplement: Supplementary file 1 — Supplementary Information [file 41467_2024_45146_MOESM1_ESM.pdf]

Supplementary Information

Perfect Circular Polarization of Elastic Waves in Solid Media

Jeseung Lee, Minwoo Kweun, Woorim Lee, Hong Min Seung & Yoon Young Kim

Supplementary Note 1: Limitations of the classical birefringence plate

A birefringent plate is a well-known device that generates circularly polarized electromagnetic waves from the incident linearly polarized electromagnetic wave [1,2]. However, the classical birefringence theory has been restricted to practically generating the circular polarization of elastic waves. In this Supplementary Note, we will explain the serious drawbacks of the elastic version of the birefringent plate.

Consider the case where a single pulse of a diagonally polarized shear wave is incident on an elastic birefringent plate sandwiched by an isotropic background solid. Let us define the mass density, stiffness, and thickness of the elastic birefringent plate as ρ_B, $\mathbf{C}_B$ and d. The mass density and stiffness of the background isotropic medium are defined as ρ_0 and $\mathbf{C}_0$. Then, the output phase difference between the SV and SH waves through the birefringent plate is calculated as

$$\Delta\phi = \angle t_{\mathrm{SV}} - \angle t_{\mathrm{SH}} = 2\pi f d \left(\frac{1}{v_{\mathrm{SV}}^{B}} - \frac{1}{v_{\mathrm{SH}}^{B}} \right), \tag{S1}$$

where t_{SV} and t_{SH} are the transmission coefficients of the SV and SH waves of the birefringent plate, respectively, and f is the frequency. The speeds of the SV and SH waves in the birefringent plate are calculated as $v_{\mathrm{SV}}^{B} = \sqrt{\frac{C_{55}^{B}}{\rho_B}}$ and $v_{\mathrm{SH}}^{B} = \sqrt{\frac{C_{66}^{B}}{\rho_B}}$, respectively.

If we want to generate the circularly polarized shear waves through the birefringent plate, the output phase $\Delta\phi$ should be $\pm\frac{\pi}{2}$. As a result, the required thickness of the birefringent plate is calculated as

$$d = \frac{1}{4f} \left| \frac{1}{v_{\mathrm{SV}}^{B}} - \frac{1}{v_{\mathrm{SH}}^{B}} \right|^{-1}. \tag{S2}$$

The result in Eq. (S2) suggests that the weak anisotropy of the birefringent plate (i.e. $v_{\mathrm{SV}}^{B} \approx v_{\mathrm{SH}}^{B}$) inevitably caused a bulky plate. Hence, the strong anisotropy of the birefringence plate is required.

On the other hand, strong birefringence should be avoided to generate the exact circular polarization (i.e., $|t_{\mathrm{SV}}| = |t_{\mathrm{SH}}|$). Applying the continuity conditions of the velocity and stress fields at the interface between the birefringent plate and background medium [3,4], the amplitude ratio of the transmitted

SV and SH waves through the birefringent plate can be calculated as

$$\frac{|t_{\mathrm{SH}}|}{|t_{\mathrm{SV}}|}=\frac{z_{\mathrm{SH}}^{B}\left(z_{\mathrm{S}}^{0}+z_{\mathrm{SV}}^{B}\right)^{2}}{z_{\mathrm{SV}}^{B}\left(z_{\mathrm{S}}^{0}+z_{\mathrm{SH}}^{B}\right)^{2}}, \tag{S3}$$

where z_{SV}^{B} and z_{SH}^{B} are the characteristic mechanical impedances of the SV and SH waves in the birefringent plate, respectively, and z_{S}^{0} is the characteristic mechanical impedance of the shear wave in the isotropic background solid. The relation in Eq. (S3) implies that the strong impedance contrast between the SV and SH waves resulted in distorted circular polarization (i.e., $|t_{\mathrm{SV}}|\neq|t_{\mathrm{SH}}|$). Furthermore, the transmitted energy intensity is very low due to the impedance mismatch between the birefringent plate and the background medium. In sum, by Eqs. (S2) and (S3), there is a trade-off between the size, accuracy, and efficiency of the classical birefringent plate.

Supplementary Note 2: Theoretical derivation of $C_{55}=C_{66}$

In this Supplementary Note, we will derive the theoretical condition of $C_{55}=C_{66}$ from condition (2) in the main text. At the incident interface of the metamaterial, the displacement vector of the incident SV wave is decomposed into the displacement vectors of the two eigenmodes, as shown in Supplementary Fig. 1. The wavenumbers (k) and polarization vectors ($\mathbf{P}=\left(P_x,P_y,P_z\right)$) of the two shear eigenmodes polarized in the *y-z* plane inside the anisotropic metamaterial are calculated by solving the governing elastic wave equation as follows [4]:

$$k^{2}=\frac{\rho\omega^{2}\left(C_{55}+C_{66}\mp\sqrt{\left(C_{55}-C_{66}\right)^{2}+4C_{56}{}^{2}}\right)}{2\left(C_{55}C_{66}-C_{56}{}^{2}\right)}, \tag{S4}$$

and

$$P_x=0, \tag{S5a}$$

$$P_y=\mp\left[1+4\left(A\pm B\right)^{-2}\right]^{-1/2}, \tag{S5b}$$

$$P_z=-\mathrm{sgn}\left(C_{56}\right)\left[\frac{1}{2}\left(B^{2}\pm AB\right)\right]^{-1/2}, \tag{S5c}$$

$$A=\frac{C_{66}-C_{55}}{|C_{56}|}\ \text{and}\ B=\sqrt{4+\left(A=\frac{C_{66}-C_{55}}{|C_{56}|}\right)^{2}}, \tag{S5d}$$

where ω is the angular frequency. In Eq. (S5), it is observed that the polarization vectors of the two eigenmodes are perpendicular to each other. When the angles formed by slow eigenmode (SE) and fast eigenmode (FE) with incident shear vertical (SV) wave are defined as θ_1 and θ_2, as shown in Supplementary Fig. 1, their sum is always 90°. At this time, if the eigenmodes are not formed in diagonal directions on the *y-z* plane, the magnitudes of two decomposed displacement vectors (denoted

as $|A_{\text{SE}}|$ and $|A_{\text{FE}}|$ in Supplementary Fig. 1) are determined differently, which leads to the generation of the distorted circular polarization through the metamaterial. Therefore, the polarization vectors of the two eigenmodes should be aligned along the upwards and downwards diagonal directions on the *y*-*z* plane. To this end, *A* and *B* in Eq. (S5) should satisfy

$$1+4(A\pm B)^{-2}=2\,, \tag{S6a}$$

$$\frac{1}{2}\left(B^2\pm AB\right)=2\,, \tag{S6b}$$

with $B=\sqrt{4+A^2}$. By solving Eq. (S6), the solution of $A = 0$ and $B = 2$ can be obtained. To satisfy this, the values of C_{55} and C_{66} must be the same. According to Eq. (S5), when the anisotropic metamaterial satisfies $C_{55}=C_{66}$ with negative C_{56}, $\mathbf{P}_{\text{SE}}=\left(0,\frac{1}{\sqrt{2}},\frac{1}{\sqrt{2}}\right)$ and $\mathbf{P}_{\text{FE}}=\left(0,-\frac{1}{\sqrt{2}},\frac{1}{\sqrt{2}}\right)$ are obtained. At this time, according to Eq. (S4), the phase speeds of slow and fast eigenmodes (defined as v_{SE} and v_{FE}, respectively) are calculated as

$$v_{\text{SE}}=\sqrt{\frac{C_{66}-|C_{56}|}{\rho}} \text{ and } v_{\text{FE}}=\sqrt{\frac{C_{66}+|C_{56}|}{\rho}}\,. \tag{S7}$$

From Eq. (S7), the wavelengths of the slow and fast eigenmodes in Eq. (2) in the main text can be obtained.

Supplementary Note 3: Theoretical derivation of Eqs. (3)-(6)

The phase-matching and impedance-matching conditions for the proposed coupled resonance can be summarized as

$$\Delta\phi_{\text{SE}}=k_{\text{SE}}d=n_{\text{SE}}\cdot\pi \quad (n_{\text{SE}}=1,2,3,\cdots), \tag{S8a}$$

$$\Delta\phi_{\text{FE}}=k_{\text{FE}}d=n_{\text{FE}}\cdot\frac{\pi}{2} \quad (n_{\text{FE}}=1,3,5,\cdots), \tag{S8b}$$

$$\rho_0 C_{66}^0=\rho\left(C_{66}+|C_{56}|\right), \tag{S8c}$$

where k_{FE} and k_{SE} are the wavenumbers of the fast and slow eigenmodes, respectively. In detail, Eq. (S8a) states half-wave phase-matching conditions for the slow eigenmode, Eq. (S8b) states quarter-wave phase-matching conditions for the fast eigenmode, and Eq. (S8c) states impedance-matching condition for the fast eigenmode. Using Eq. (S4), the wavenumbers of the fast and slow eigenmode are calculated as

$$k_{\text{FE}}=2\pi f\sqrt{\frac{\rho}{C_{66}+|C_{56}|}}\,, \tag{S9a}$$

$$k_{SE} = 2\pi f \sqrt{\frac{\rho}{C_{66} - |C_{56}|}}, \tag{S9b}$$

where f is the frequency. By substituting Eq. (S9) into Eqs. (S8a) and (S8b), we can obtain

$$\sqrt{\frac{\rho}{C_{66} + |C_{56}|}} = \frac{n_{FE}}{4fd}, \tag{S10a}$$

$$\sqrt{\frac{\rho}{C_{66} - |C_{56}|}} = \frac{n_{SE}}{2fd}. \tag{S10b}$$

To sum up, there are a total of three theoretical conditions, Eq. (S8c), Eq. (S10a), and Eq. (S10b), that a total of three unknown physical properties, ρ, $C_{66}(=C_{55})$, and C_{56}, should satisfy. As a result, we can explicitly determine the physical properties of the anisotropic metamaterial as follows:

$$\rho = \frac{1}{4f \cdot d} \cdot n_{FE} \cdot \sqrt{\rho_0 C_{66}^0}, \tag{S11a}$$

$$C_{55} = C_{66} = \frac{1}{2} f \cdot d \cdot n_{FE} \left(\frac{4}{n_{FE}^2} + \frac{1}{n_{SE}^2} \right) \sqrt{\rho_0 C_{66}^0}, \tag{S11b}$$

$$|C_{56}| = \frac{1}{2} f \cdot d \cdot n_{FE} \left(\frac{4}{n_{FE}^2} - \frac{1}{n_{SE}^2} \right) \sqrt{\rho_0 C_{66}^0}, \tag{S11c}$$

which is exactly the same result in Eqs. (3)-(6) in the main text.

Supplementary Note 4: Frequency responses

In Supplementary Fig. 2, we calculated the transmission and reflection spectra of the proposed metamaterial considered in Fig. 1 in the main text. At the resonance frequency of 100 kHz, the magnitude balance condition ($|t_{SV}|^2 = |t_{SH}|^2 = 0.5$) and 90° out-of-phase condition ($|\angle t_{SV} - \angle t_{SH}| = 0.5\pi$) were achieved with the longitudinal-free conditions ($|t_L|^2 = |r_L|^2 = 0$) and reflection-free conditions ($|r_{SV}|^2 = |r_{SH}|^2 = 0$). Interestingly, by adjusting the frequency to a multiple of the resonance frequency, the anisotropic metamaterial can generate all of the LCS, SH, RCS, and SV waves from the incident SV wave with 100% energy efficiency. Supplementary Fig. 3 shows the output polarization through the metamaterial at integer multiples of the resonance frequency. We note that the SV-to-LCS, SV-to-SH, SV-to-RCS, and SV-to-SV polarization conversion is realized at the frequencies of 100, 200, 300, and 400 kHz, respectively. Furthermore, the output polarization remains almost the same as at the target frequency with ±5 kHz frequency shifts, confirming the robust performance of the proposed metamaterial.

Supplementary Note 5: Types of polarization conversion

When an SV wave is incident on the anisotropic metamaterial that satisfies Eqs. (3)-(6) in the main

text, transmission coefficients of SV and SH waves are calculated as follows:

$$t_{\mathrm{SV}}=\left[\frac{(-1)^{n_{\mathrm{SE}}}}{2}+\frac{(-1)^{\frac{n_{\mathrm{FE}}+1}{2}}}{2}i\right]e^{i\phi_{\mathrm{S}}}, \tag{S12a}$$

$$t_{\mathrm{SH}}=\mathrm{sgn}(C_{56})\left[\frac{(-1)^{n_{\mathrm{SE}}+1}}{2}+\frac{(-1)^{\frac{n_{\mathrm{FE}}+1}{2}}}{2}i\right]e^{i\phi_{\mathrm{S}}}, \tag{S12b}$$

where ϕ_{S} is the phase correction of the shear wave for wave propagation through the metamaterial. In order to generate an LCS (RCS) wave, a phase difference between the transmitted SV and SH wave should satisfy $\angle t_{\mathrm{SV}}-\angle t_{\mathrm{SH}}=0.5\pi$ ($\angle t_{\mathrm{SV}}-\angle t_{\mathrm{SH}}=-0.5\pi$). By Eq. (S12), if the metamaterial exploits the fundamental coupled resonance ($n_{\mathrm{FE}}=n_{\mathrm{SE}}=1$), an LCS (RCS) wave is transmitted from the metamaterial with a negative (positive) C_{56}.

The type of the generated circular polarization can be fully controlled by the polarization (SV or SH) and propagation direction (+*x* or –*x*-direction) of the incident linearly polarized shear wave because of the microstructural symmetry of the metamaterial. Supplementary Fig. 4 shows that, under the SH wave incidence, the RCS waves were transmitted through the same metamaterial in Fig. 2 at 100 kHz. Furthermore, under the SV (or SH) wave incidence from behind the metamaterial in the –*x*-direction, the type of the generated circular polarization becomes right-handed (or left-handed), as shown in Supplementary Fig. 5. It means that it is possible to realize all four types of linear-to-circular polarization conversion (SV-to-LCS, SV-to-RCS, SH-to-LCS, and SH-to-RCS) using only one elaborately designed metamaterial.

Supplementary Note 6: Applicability and limitation of the proposed method

In the main text, we chose aluminum as the target isotropic background material and 100 kHz as the target frequency. However, the proposed coupled resonance is applicable to other isotropic background materials and frequencies. Supplementary Table 2 provides the required physical properties of the anisotropic medium to realize the coupled resonance at various target frequencies (50, 100, and 150 kHz) when the target materials are aluminum (metal) and PEEK (plastic). The density of aluminum and PEEK is 2700 kg m^3 and 1320 kg m^3, and the shear stiffness of aluminum and PEEK is 26.3 GPa and 1.51 GPa. Without loss of generality, the thickness of the utilized anisotropic medium with $n_{\mathrm{SE}}=n_{\mathrm{FE}}=1$ is assumed to be $d=0.01\,\mathrm{m}$.

The effectiveness of the proposed method is contingent on the feasibility of manufacturing metamaterial microstructures, which varies based on the target material and frequency. For instance, higher target frequencies necessitate increasingly smaller subwavelength-scale microstructures. However, current manufacturing technologies, like the drilling procedure employed in our experiments, may not be capable of producing these intricate microstructures.

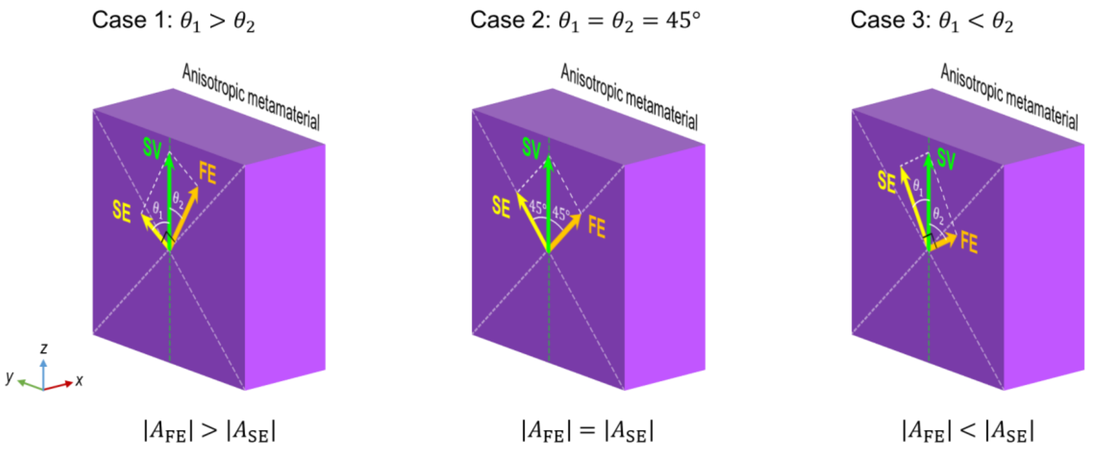

Supplementary Fig. 1. Modal decomposition at the incident interface of the anisotropic metamaterial. An incident shear vertical wave (SV, green) is decomposed into a slow eigenmode (SE, yellow) and a fast eigenmode (FE, orange) at an incident interface of the anisotropic metamaterial.

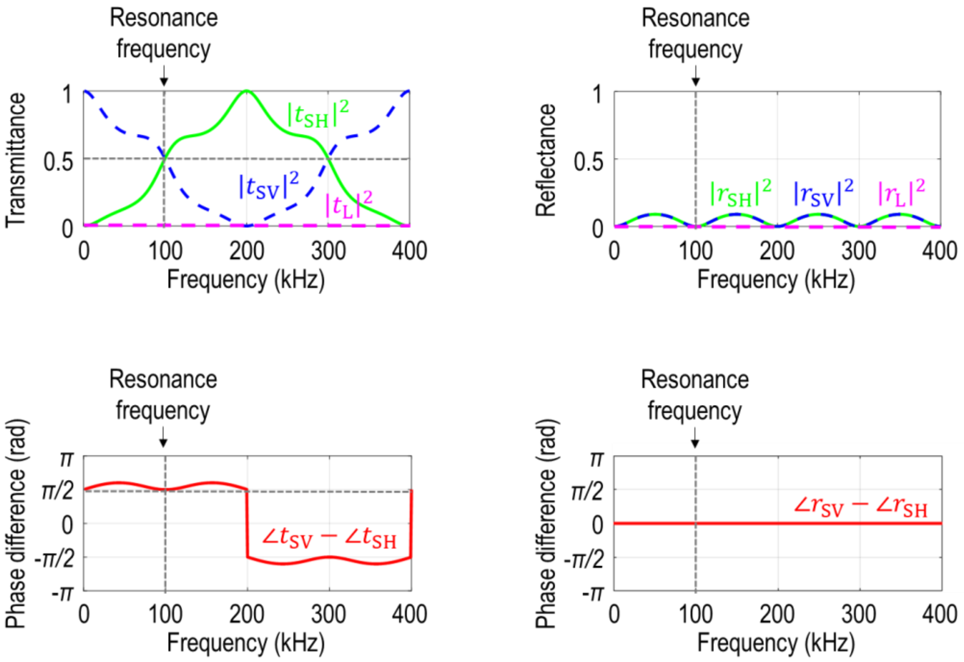

Supplementary Fig. 2. Frequency responses of scattering parameters of the anisotropic metamaterial. Transmission and reflection coefficients of L (magenta), SV (blue), and SH (green) waves under the SV wave incidence to the anisotropic metamaterial are presented as a function of a frequency. The phase differences (red) between SV and SH waves are also presented.

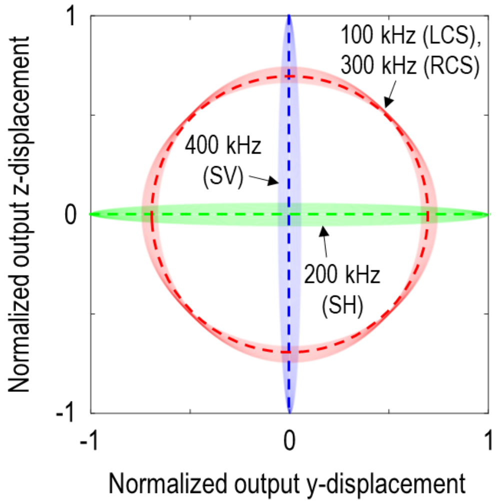

Supplementary Fig. 3. Frequency responses of the output polarization through the anisotropic metamaterial. The output polarization is calculated at integer multiples of the resonance frequency (specifically, at 100, 200, 300, and 400 kHz). The output *y*- and *z*-displacements are normalized by the input displacement amplitude. The deviation of the output polarization is also presented in the frequency range of around 5 kHz.

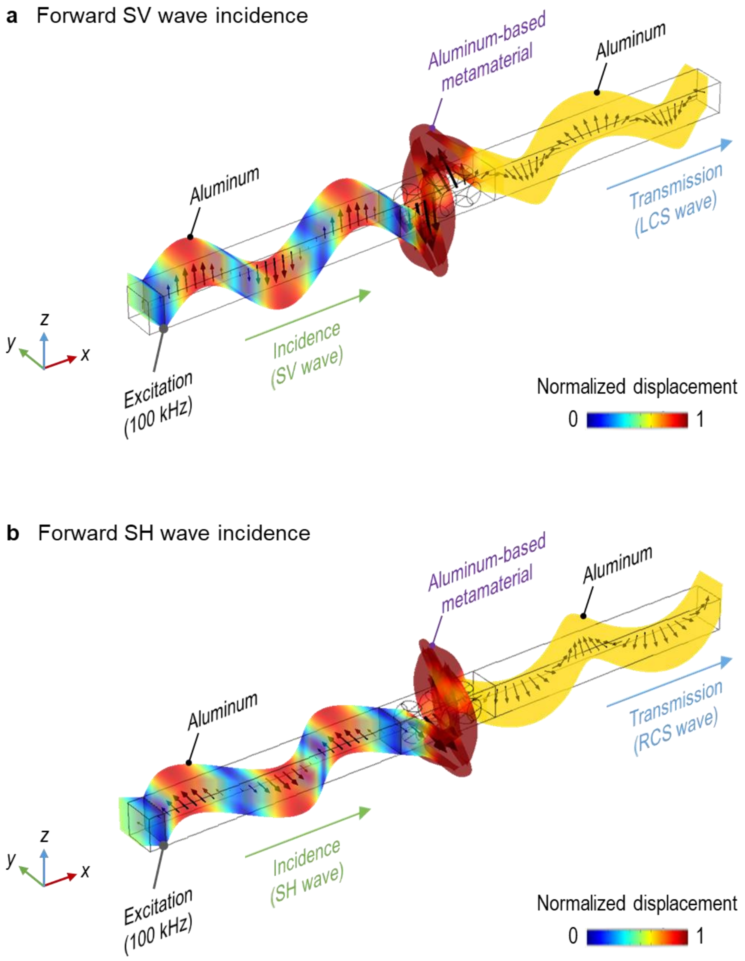

Supplementary Fig. 4. 3D time-harmonic numerical simulation results under forward SV and SH wave incidence. a-b, SV and SH waves are incident in the positive x-direction to the designed metamaterial and converted into LCS and RCS waves at 100 kHz, respectively.

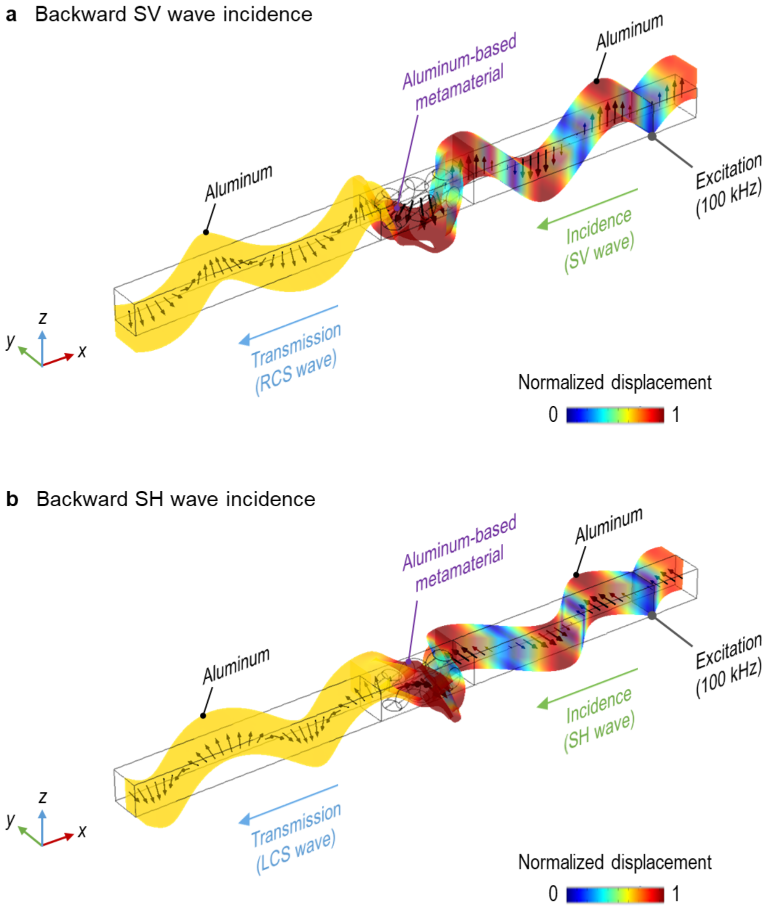

Supplementary Fig. 5. 3D time-harmonic numerical simulation under backward SV and SH wave incidence. a-b, SV and SH waves are incident in the negative x-direction to the designed metamaterial and converted into RCS and LCS waves at 100 kHz, respectively.

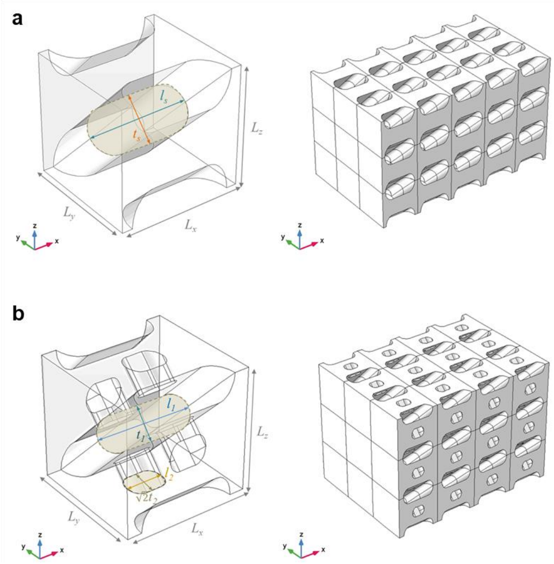

Supplementary Fig. 6. Microstructure candidates for realizing the proposed coupled resonance. **a,** Rounded cuboidal void microstructure. **b,** Crossed rounded cuboidal void microstructure.

Supplementary Table 1. Optimized geometric parameters of the unit cell

L_x [mm]	L_y [mm]	L_z [mm]	r_1 [mm]	r_2 [mm]	r_3 [mm]	L [mm]
19.89	6.173	6.173	1.428	1.725	1.470	5.430

Supplementary Table 2. The required physical properties of the anisotropic medium to realize the coupled resonance at various target materials and frequencies. The thickness of the utilized anisotropic medium with $n_{\mathrm{SE}} = n_{\mathrm{FE}} = 1$ is assumed to be $d = 0.01\,\mathrm{m}$.

Background: Aluminum	ρ [kg m^3]	C_{55} [GPa]	C_{66} [GPa]	C_{56} [GPa]
50 kHz	4213.4	10.5	10.5	-6.30
100 kHz	2106.7	21.1	21.1	-12.6
150 kHz	1404.5	31.6	31.6	-19.0
**Background: PEEK**	ρ [kg m^3]	C_{55} [GPa]	C_{66} [GPa]	C_{56} [GPa]
50 kHz	705.9	1.76	1.76	-1.06
100 kHz	353.0	3.53	3.53	-2.12
150 kHz	235.3	5.29	5.29	-3.18

Supplementary References

[1] Yariv, A. & Yeh, P. *Optical Waves in Crystals* (Wiley, 1984).

[2] Pedrotti, F. L., Pedrotti, L. M. & Pedrotti, L. S. *Introduction to Optics* (Cambridge University Press, 2017).

[3] Kinsler, L. E. et al. *Fundamentals of Acoustics* (John Wiley & Sons, 2000).

[4] Auld, B. A. *Acoustic Fileds and Waves in Solids* (Krieger Publishing Company, 1990).
