# Supplementary material for: Perfect circular polarization of elastic waves in solid media
Source: Nat Commun. 2024 Feb 12;15:992. doi: 10.1038/s41467-024-45146-w (PMC10861468; doi:10.1038/s41467-024-45146-w)
Supplement: Supplementary file 3 — Description of Additional Supplementary Files [file 41467_2024_45146_MOESM3_ESM.pdf]

**Description of Additional Supplementary Files**

**File Name:** Supplementary Movie 1
**Description:** A video demonstration of the coupled resonance theory. The total displacement field (green) inside the anisotropic metamaterial is decomposed into the displacement fields of the half-wavelength-matched slow eigenmode (yellow) and quarter-wavelength-matched fast eigenmode (orange).

**File Name:** Supplementary Movie 2
**Description:** A video demonstration of the spatiotemporal displacement field inside the designed unit cell. The SV wave is incident on the unit cell in the x-direction at 100 kHz.

**File Name:** Supplementary Movie 3
**Description:** A video demonstration of the time-transient numerical simulation with the designed unit cell. The SV wave is incident on the unit cell in the x-direction at 100 kHz.
